# A Three-Dimensional Comprehensive Numerical Model of Ion Transport during Electro-Refining Process for Scrap-Metal Recycling

**DOI:** 10.3390/ma15082789

**Published:** 2022-04-11

**Authors:** Chang Liu, Guangqiang Li, Lifeng Zhang, Qiang Wang, Qiang Wang

**Affiliations:** 1 The State Key Laboratory of Refractories and Metallurgy, Wuhan University of Science and Technology, Wuhan 430081, China; liuchang2021@wust.edu.cn (C.L.); liguangqiang@wust.edu.cn (G.L.); 2 Key Laboratory for Ferrous Metallurgy and Resources Utilization of Ministry of Education, Wuhan University of Science and Technology, Wuhan 430081, China; 3 School of Mechanical and Materials Engineering, North China University of Technology, Beijing 100144, China; zhanglifeng@ncut.edu.cn; 4 Key Laboratory of Electromagnetic Processing of Materials (Ministry of Education), Northeastern University, Shenyang 110819, China; wangq@mail.neu.edu.cn

**Keywords:** metal recycling, electro-refining process, ion transport, convection, electro-migration, computational fluid dynamics

## Abstract

A transient three-dimensional comprehensive numerical model was established to study ion transport caused by diffusion, convection, and electro-migration in the electro-refining process for scrap-metal recycling. The Poisson–Nernst–Planck equations were used to define ion transport within the electrolyte, while the Naiver–Stokes equations and the energy equation were employed to describe fluid flow and heat transfer. In addition, the Butler-Volmer formulation was used to represent the kinetics of the electrochemical reaction. The comparison between the measured and simulated data indicates the reliability of the model. Under the action of diffusion and electro-migration, the positive copper ion moves from the anode to the cathode, while the negative sulfate ion migrates in the opposite direction. The distribution of the ion concentration, however, greatly changes if the fluid flow is taken into account. The ion concentration around the anode and the rate of the electrochemical reaction that occurs at the anode surface are reduced by the fluid flow. The proposed computational framework offers a valuable basis for future research and development in the field of scrap-metal recycling technology.

## 1. Introduction

Metals are crucial to the development of our global society, as they are essential, are versatile, and can be used as the key components of a large number of products such as cars, buildings, ships, and railways [1]. Today, the manufacture of these products mainly depends on the availability of newly extracted metals, which are typically mined from ores. However, raw material on the earth that can be used for smelting is limited. Due to the diminishing supply of raw material, metal recycling is, therefore, becoming increasingly important [2]. Metal recycling not only helps lessen the consumption of raw material, but also reduces energy usage and pollution. With the recycling of every 1 kg of scrap steel, savings of 1.4 kg of iron ore, 1.5 kg of CO_2_ emissions, and 13.4 MJ of primary energy can be fulfilled. This is equivalent to 73%, 64%, and 90%, respectively, when compared to 100% primary production [3].

Electro-refining technology is more and more used currently for recycling discarded metal since the electro-refining process can be designed to handle a wide change in the quality of the base scrap metal and is able to provide a high purity of end-product material [4,5,6]. During the process, an electrochemical reaction takes place. The crude metal, which serves as an anode, loses electrons and generates metal ions that enter the electrolyte. Through the ways of diffusion, convection, and electro-migration, the metal cations then move toward the cathode, which is a piece of pure metal, and deposit as pure metal on the surface of the cathode. By controlling the process parameters, the impurities in the anode that are less electropositive than the metal being deposited would not lose electrons and accumulate at the bottom of the electrolyte. The more electropositive impurities would become ions and stay suspended in the electrolyte.

Numerous studies have been conducted on the electro-refining process of many scrap metals and some potential applications [7,8,9]. Nevertheless, some metals, especially rare earth and reactive metals, have multivalent states with little difference in the electrochemical stability. Two or more separate valence species may coexist in the electrolyte, which greatly reduces the current efficiency of the electro-refining process [10]. In order to improve the current efficiency, it is desirable to increase the electrical conductivity of the electrolyte in the cell [11]. Aji et al. investigated the effect of free acid, silver, copper, and lead concentrations on the electrical conductivity of the electrolyte during silver electro-refining [12]. The results showed that an increase in all ion concentrations had a positive effect on silver electrolyte conductivity. Moreover, free acid concentration was found to have the most significant impact on the electrical conductivity of the electrolyte. Ion transport behavior is regarded as playing a critical role in determining the electrical conductivity of the electrolyte. Hence, it is of great interest to understand ion transport within the electrolyte, which includes three different mechanisms: diffusion, convection, and electro-migration [13].

Numerical simulation has gained increased attention in recent years, due to its ability to clarify the transport phenomena in the electrolytic process, which cannot be revealed by experiments at the current time. Some investigations [14,15,16] focused on the theoretical mechanism of the reaction, permeation, etc., which provide the theoretical support for the simulation of the ion transport process combined with the actual three-dimensional flow. Wu and Chen developed a two-dimensional axisymmetric mathematical model to study the movement of cooper ions in a batch rotating cylinder electrode system [17]. The Poisson–Nernst–Planck (PNP) and the Butler–Volmer (BV) equations were used to describe ion transport and the kinetics of the electrochemical reaction, respectively. In addition, the Naiver–Stokes (NS) equations were employed to take into account the flow of the electrolyte. The results indicated that the mass transfer of the copper ion was influenced by fluid flow, which caused an accumulation of copper ions at the top corner of the cell. Park et al. compared the predictive ability of the two-dimensional and three-dimensional electro-refining numerical models [18]. Their research found that the two-dimensional model showed some statistical noise at high applied current densities. The three-dimensional model, however, could predict the overall tendency of the current distribution along with the cathode with acceptable accuracy. Choi et al. analyzed the electro-fluid-dynamic behavior in a molten-salt electro-refining process by the coupling of a three-dimensional computational fluid dynamics module and a one-dimensional electrochemical reaction analysis module [19]. The distributions of the local ion concentration and local multispecies current density were demonstrated. Nevertheless, the electrolyte was assumed to be isothermal in all simulation scenarios mentioned above. It has been known in the electrochemistry research community that heat transfer plays a vital role in ion transport. For instance, Caglayan et al. established a three-dimensional numerical model for exploring the effect of electrolyte temperature on the electrochemical reaction rate in a fuel cell [20]. They observed that the output power density of the fuel cell increased from 0.26 W/cm^2^ to 0.42 W/cm^2^ when the electrolyte temperature ranged from 373 K to 453 K. They attributed the increase to the faster reaction kinetics and higher membrane proton conductivity at higher temperatures.

In the present work, a transient three-dimensional numerical model of the electro-refining process was created to consider the coupled physical phenomena. These are, among others, multi-ion transport processes inside the electrolyte, influenced by the electric field, fluid flow, and heat transfer, as well as electrochemical reactions occurring at the electrode/electrolyte interfaces. All three ion-transport mechanisms in the electrolyte, i.e., diffusion, convection, and electro-migration, are accounted for in the model. It is emphasized that none of these effects is neglected. The finite volume approach was utilized to simultaneously solve the PNP, the NS, the energy, and the BV equations. In addition, experiments were carried out to validate the mathematical model developed in the present work.

## 2. Experiment

**Figure 1:**
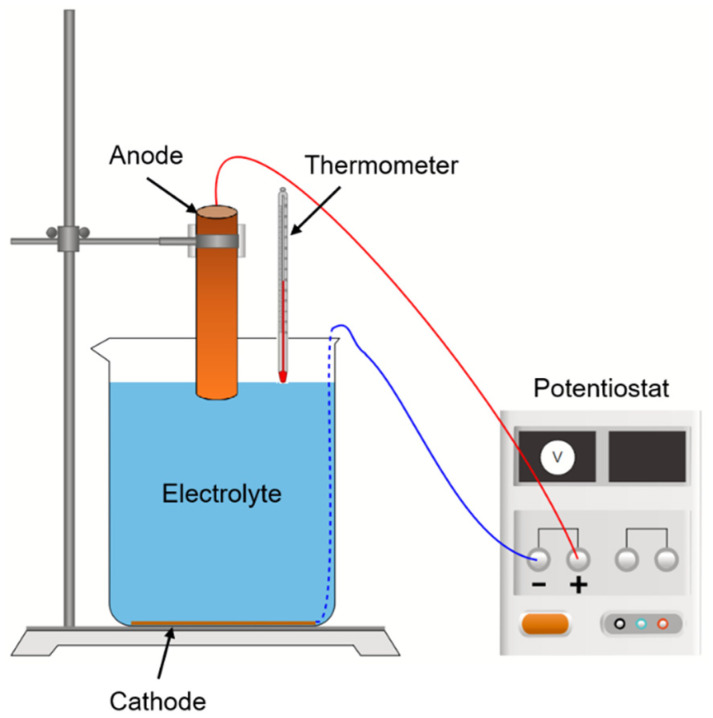
Schematic of the experimental setup.

Figure 1 shows the schematic diagram of the experimental setup. A 99.9% purity copper bar (Shanghai Xiongyu Industrial Co., Ltd., Shanghai, China) with 20 mm diameter served as the anode, while a 99.9% purity copper disc, (Shanghai Xiongyu Industrial Co., Ltd., Shanghai, China) with 55 mm diameter and 0.1 mm thickness, was employed as the cathode. The electrolyte in the beaker was 40 g/L CuSO_4_ solution (Sinopharm Chemical Reagent Co., Ltd., Shanghai, China) with 62 mm height, and the temperature was about 288 K. The anode was inserted into the electrolyte at around 5 mm immersion depth. The cathode copper plate was connected to the potentiostat (Beijing hengruixinda Technology Co., Ltd., Beijing, China) through a welded copper wire. Before the electrolysis begins, both electrodes and the container were soaked in deionized water and placed in an ultrasonic cleaning machine (Zhongkeyi (Beijing) Instrument Co., Ltd., Beijing, China) for 10 min to remove surface impurities, and finally dried by nitrogen gas.

Afterward, a constant voltage of 2.2 V was applied across the anode and the cathode by a potentiostat, and the electrolytic process continued for 10 min. In order to detect variation of the copper ion concentration, the treated electrolyte was sampled during the electrolytic process. To minimize the influence of sampler insertion on the flow, samples were taken at a shallow depth of electrolyte. The copper ion concentration was then analyzed, using inductively coupled plasma optical emission spectrometry. In addition, a digital thermometer (Shanghai Aladdin Biochemical Technology Co., Ltd., Shanghai, China) was used to measure the temperature of the electrolyte.

## 3. Mathematical Model

### 3.1. Assumptions

The following assumptions were considered to reasonably simplify the numerical model:

(1) The ambient air and the electrodes were disregarded in the computational domain.
(2) The electrolyte was assumed to be incompressible Newtonian fluid. The density was influenced by temperature and ion concentration, and other properties, such as the electrolyte conductivity and ion diffusion coefficient, were considered constant.
(3) The electrochemical reactions of other metal ions were ignored with the exception of the reactions that involve copper ion, since the purity of the copper electrode was quite high.

### 3.2. Fluid Flow and Heat Transfer

The continuity and time-averaged NS equations were invoked to express the movement of the electrolyte in the container [21].

(1) $$\frac{\partial \rho }{\partial t}+\nabla \cdot \left(\rho \vec{v}\right)=0,$$

(2) $$\frac{\partial \left(\rho \vec{v}\right)}{\partial t}+\rho \vec{v}\cdot \nabla \vec{v}=-\nabla p+\nabla \cdot \left(\mu_{eff}\nabla \vec{v}\right)+{\vec{F}}_{s}+{\vec{F}}_{t},$$

where *ρ* is the density of the electrolyte, *t* is the time, $\vec{v}$ is the velocity, *p* is the pressure, $\mu_{eff}$ is the dynamic viscosity of the electrolyte, and $\vec{F}{}_{s}$ and $\vec{F}{}_{t}$ are the solutal and the thermal buoyancy forces determined by the Boussinesq approximation.

According to observations in the experiment, the natural convection of the electrolyte was presumed to be weak. Here, it was assumed that the maximal speed of the electrolyte was on the order of magnitude of 10^−2^ m/s. The inner diameter of the container, 55 mm, was defined as the characteristic length. The largest Reynolds number was consequently around 153. The flow regime of the electrolyte in the container was laminar rather than turbulent.

The following energy equation could be solved for the thermal field of the electrolyte [22]:

(3) $$\frac{\partial \left(\rho h\right)}{\partial t}+\nabla \cdot \left(\rho \vec{v}h\right)=\nabla \cdot \left(k_{eff}\nabla T\right)+Q_{F}+Q_{J},$$

where *h* is the sensible enthalpy of the electrolyte, $k_{eff}$ is the effective thermal conductivity, *T* is the temperature, and the last two terms $Q_{F}$ and $Q_{J}$ represent the heat source created by the electrochemical reaction and the electric current, respectively.

### 3.3. Kinetics of Electrochemical Reaction

During the electrolytic process, two electrochemical reactions could be observed at the anode/electrolyte and the cathode/electrolyte interfaces.

Anode/electrolyte interface:

(4) $$\mathrm{Cu}\to {\mathrm{Cu}}^{2+}+{2e}^{-};$$

Cathode/electrolyte interface:

(5) $${\mathrm{Cu}}^{2+}+{2e}^{-}\to \mathrm{Cu}.$$

The rate of charge transfer reaction for the two electrochemical reactions was modeled by the BV formulation [23,24].

(6) $$i=i_{0}\left[exp\left(-\frac{\alpha z_{i}F}{RT}\eta \right)-\frac{c_{i}}{c_{i,0}}exp\left(\frac{\left(1-\alpha \right)z_{i}F}{RT}\eta \right)\right],$$

where *i* is the current density, $i_{0}$ is the exchange current density, *α* is the transfer coefficient, $z_{i}$ is the charge number of the *i*-th ion, *F* is the Faraday law constant, *R* is the gas constant, $c_{i}$ is the mass fraction of the *i*-th ion, and $c_{i,0}$ is the initial mass fraction of the *i*-th ion. η is the overpotential, created under the action of the applied voltage, which is calculated as

(7) $$\eta =\phi_{s}-\phi_{l}-{Ε}_{eq},$$

where $\phi_{s}$ is the potential on the electrode surface, $\phi_{l}$ is the potential in the electrolyte, and $E_{eq}$ is the equilibrium potential. According to the BV formulation, the driving force for the electrochemical reaction is the potential difference across the electrode/electrolyte interface. The potential in the medium can be derived from the charge conservation law [25].

(8) $$\nabla \cdot \vec{i}=0.$$

In the electrode, the current density and the potential gradient are related through Ohm’s law.

(9) $$\vec{i}=-\sigma \nabla \phi_{s},$$

where σ is the electrical conductivity. In the electrolyte, the current density is determined by the fluxes of the ions.

(10) $$\vec{i}=F\sum_{i}z_{i}{\vec{N}}_{i},$$

where ${\vec{N}}_{i}$ is the total flux of the *i*-th ion. The PNP equations were then employed to describe the fluxes of ions through the electrolyte, which was subjected to an electric field [26,27,28].

(11) $$-\Delta \phi_{l}=\frac{F}{\varepsilon_{0}\varepsilon_{r}}\sum z_{i}c_{i},$$

(12) $$\frac{\partial c_{i}}{\partial t}=-\nabla \cdot {\vec{Ν}}_{i},$$

(13) $${\vec{Ν}}_{i}=\vec{v}c_{i}-D_{i}\nabla c_{i}-\frac{Fz_{i}D_{i}\nabla \phi_{l}}{RT}c_{i},$$

where $\varepsilon_{0}$ and $\varepsilon_{r}$ are the vacuum dielectric permittivity and the relative dielectric permittivity, respectively, and $D_{i}$ is the diffusion coefficient of the *i*-th ion. Additionally, the three terms on the righthand side of Equation (13) indicate the driving forces for the fluxes of the ions: convection, diffusion, and electro-migration, respectively. Furthermore, the concentrations of each ion were confined according to the electroneutrality law.

(14) $$\sum_{i}z_{i}c_{i}=0.$$

Electrochemistry contributes two distinct sources of heat to the energy equation as stated above. The first term, $Q_{F}$, represents the electrochemical reaction heat, and is created by the charge transfer reaction at the interfaces of the anode/electrolyte and the cathode/electrolyte. The second term, $Q_{J}$, is the Joule heat due to the motion of ions in the electrolyte.

(15) $$Q_{F}=\sum_{i}\left|i\cdot \eta \right|.$$

(16) $$Q_{J}=\sigma {\left|\nabla \phi_{l}\right|}^{2}.$$

### 3.4. Boundary Conditions

A constant voltage, 2.2 V, was imposed at the electrode tip, while the voltage was set to zero at the cathode. The top surface and the wall were assumed to be electrically insulated. The initial concentration and temperature of the CuSO_4_ electrolyte were 40 g/L and 288 K, respectively.

Since the electrolytic process was performed in the atmosphere, the top surfaces of the electrolyte, the wall, and the bottom of the container were assumed to exchange heat with the surrounding air by natural convection. The convective heat transfer coefficient of these walls was adjusted according to the experiment carried out in the present work.

In order to better represent the flow at the free surface, a zero shear stress was employed on the top surface of the electrolyte, while other walls were assumed to follow the no-slip boundary condition.

## 4. Numerical Procedure

**Figure 2:**
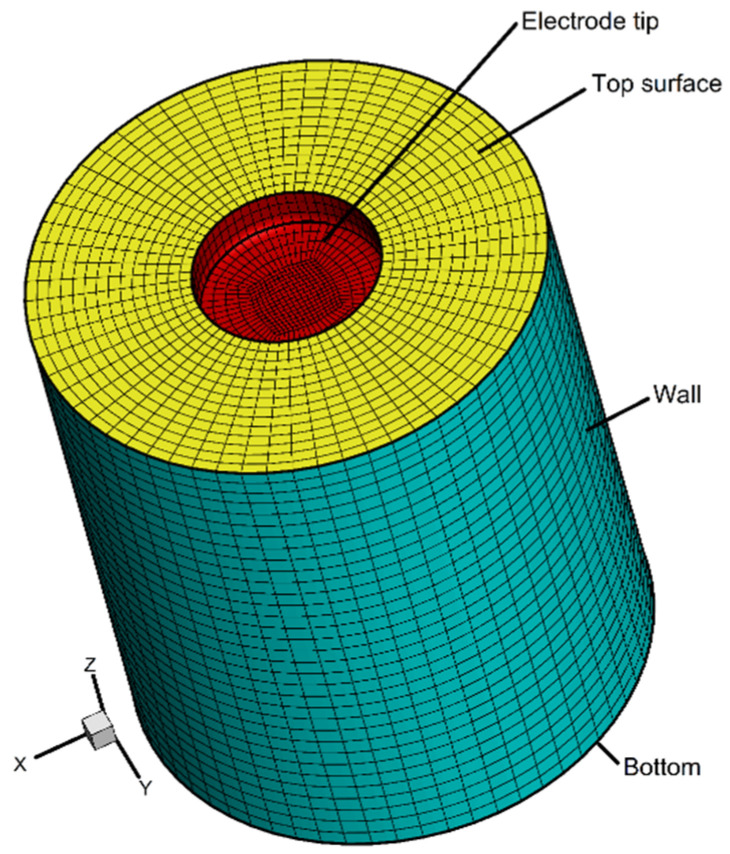
Mesh used in the simulation and boundaries.

The computational domain was discretized with a nonuniform structured mesh. The state variables, such as ion content and potential, dramatically changed within the electrochemical boundary layer, which rose near the electrodes. A refined mesh was, thus, created in these regions because of the presence of the high gradient. The grid independence was studied using three different mesh sizes with 1,750,000, 1,104,000, and 820,000 cells. From the simulation results, it was found that the relative deviation of copper ion content between the first and second mesh systems was about 2.3%, while the relative deviation was around 5.1% between the second and third mesh systems. Moreover, the value of y+, which was suggested to be lower than 1, ranged from 0.3 to 0.7 of the three generated meshes. Considering the computational accuracy and efficiency, the mesh with 1,104,000 cells was retained for the further simulated results. Figure 2 shows the mesh and boundaries. Here, the grid pattern is coarsely displayed for ease of visualization.

In the present work, the commercial software ANSYS-FLUENT (14.5, 2012, FLUENT Inc. Company, New York, NY, USA), based on the finite volume technique, was adopted to transform and solve the governing equations of the electrical, velocity, temperature, and ion concentration fields [29]. The development of the ion transport module and the introduction of the BV equation were accomplished by homemade codes. The SIMPLE algorithm was used to deal with the pressure–velocity coupling. To obtain higher accuracy, the second-order upwind scheme was chosen for the discretization of all equations. The solution was iterated until convergence was achieved when all normalized unscaled residuals were less than 10^−6^. A timestep of 10^−4^ s was used for all simulation scenarios. A typical scenario spent approximately 560 CPU hours, using 16 cores at 4.2 GHz.

## 5. Results and Discussion

### 5.1. Electrical Field and Heat Distribution

**Figure 3:**
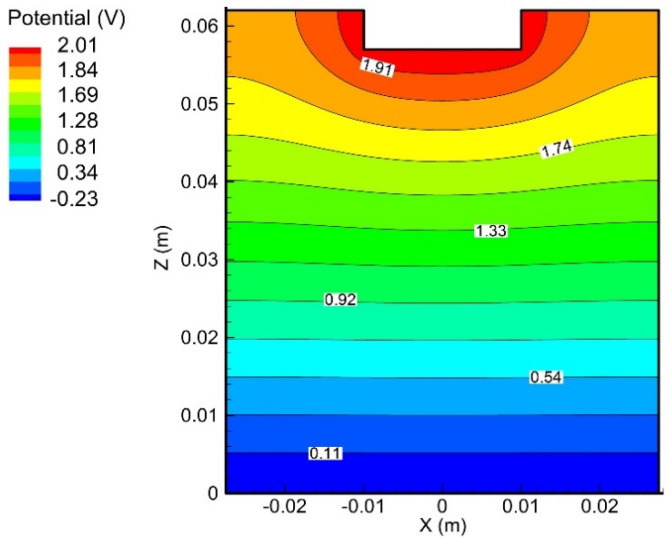
Distribution of electrolyte potential in the electrolyte at 300 s.

Figure 3 displays the distribution of the electrical potential in the electrolyte. The potentials close to the anode and the cathode were 2.01 V and −0.23 V, respectively. Due to the overpotential, the potentials were lower than the corresponding applied voltages.

**Figure 4:**
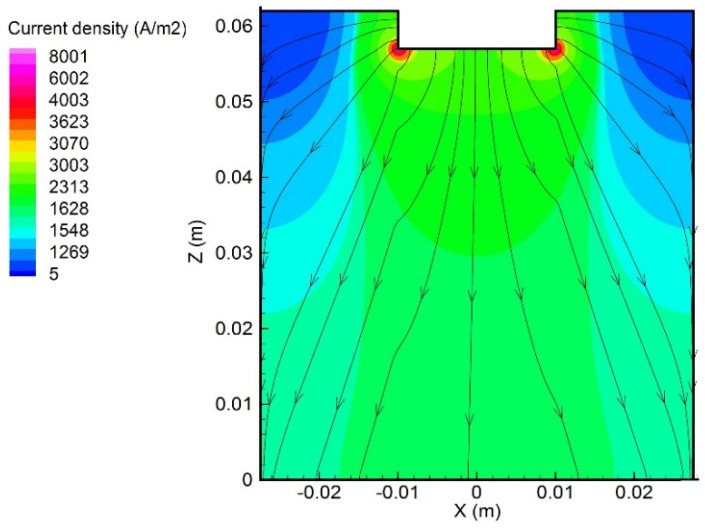
Distributions of current streamlines and current density in the electrolyte at 300 s.

Figure 4 shows the current streamlines and the current density distribution in the electrolyte at 300 s. Once the current entered the electrolyte from the anode tip, it spreads around and then moved downward to the cathode. The highest current density was observed in the electrolyte closest to the outermost part of the anode tip, and the lowest current density was located at the outer side of the top layer of the electrolyte.

**Figure 5:**
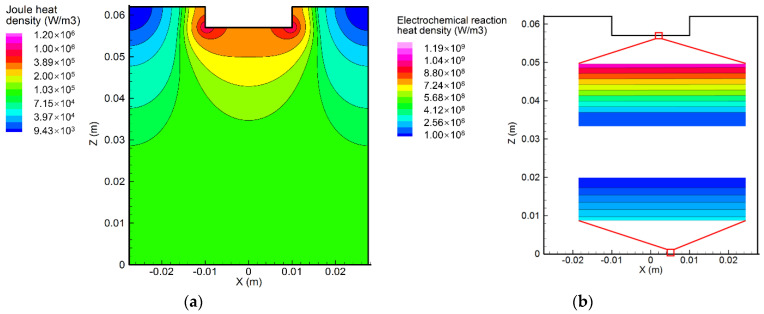
Distribution of heat density (**a**) created by the current, and (**b**) created by the electrochemical reaction.

Figure 5a,b demonstrate the heat density distribution in the electrolyte induced by the current and the electrochemical reaction, respectively. The Joule heating is determined by the current density and the electrical resistivity. As a result, the distribution of the Joule heating density was similar to that of the current density. It can be seen that the location where the maximal Joule heating density occurred was the same as that of the maximum current density. Due to a sparser current streamline, the minimum Joule heating density was also found at the outer side of the top electrolyte. On the other hand, the electrochemical reaction could also generate significant amounts of heat within the shallow layer of the electrolyte attached to the electrodes. Furthermore, the heat density caused by the electrochemical reaction was around 1000 times greater than that induced by the current.

### 5.2. Flow Field and Temperature Distribution

**Figure 6:**
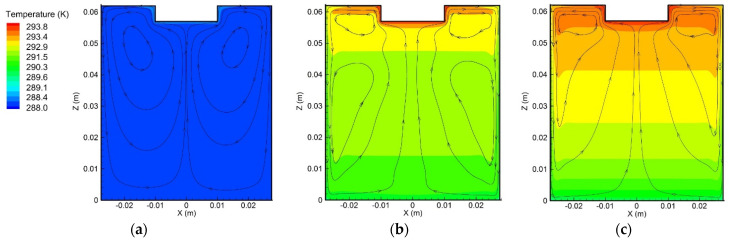
Variations of the flow pattern and temperature distribution over time: (**a**) *t* = 10 s, (**b**) *t* = 300 s, and (**c**) *t* = 600 s. The maximum velocity was about 0.008 m/s.

Figure 6 represents the variation of the flow pattern and temperature distribution over time. With more heat, the electrolyte around the anode tip gradually became hotter, resulting in the ascension of the electrolyte. The electrolyte then moved along the radial direction toward the outer edge and, from there, down the container wall. The heat in the vicinity of the container wall was removed by the ambient air, causing the electrolyte to sink. The downward streams reversed when they reached the container bottom, and then converged at the center, developing a larger stream, before finally moving upward, creating a clockwise circulation, as shown in the right side of Figure 6a. Since the temperature of the top electrolyte increased, the larger vortex was then divided into two smaller vortices, as shown in Figure 6b. The lower vortex gradually grew bigger over time and swallowed the upper smaller vortex, as shown in Figure 6c. Due to more heat, the upper electrolyte was hotter than the lower one, and the highest temperature, about 294 K, was found around the anode tip. Furthermore, the maximum velocity of the electrolyte was approximately 0.008 m/s; thus, the largest Reynolds number was equal to 191.25, indicating that the flow of the electrolyte was far from turbulent and verifying the choice of the laminar flow computational method.

**Figure 7:**
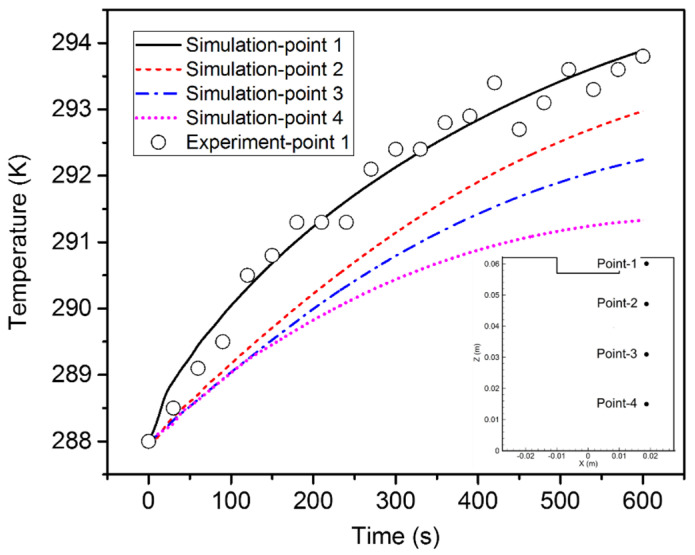
Variation of the temperature over time at the four points.

Figure 7 demonstrates the variation of the temperature of four points in the electrolyte over time. The electrolyte temperature gradually became higher as expected, with the highest temperature at point 1. The temperature measurement position can be found in Figure 1, which is near point 1 in the schematic in Figure 7. The temperature of point 1 was measured during the experiment and compared with the simulated value. A reasonable agreement was obtained, suggesting the reliability of the developed model.

### 5.3. Distribution of Ion Concentration

**Figure 8:**
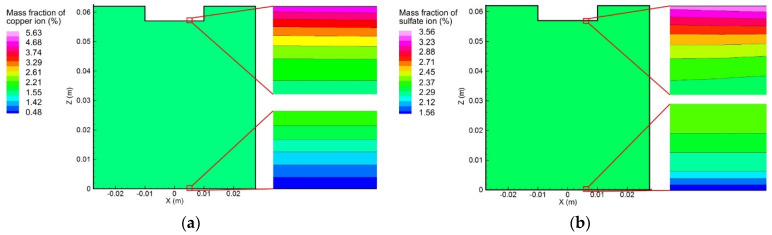
Distribution of ion concentration at 10 s: (**a**) copper ions; (**b)** sulfate ions.

Figure 8 represents the distribution of the ion mass fraction at 10 s. The initial mass fractions of the copper and sulfate ions in the electrolyte were 1.54% and 2.31%, respectively. As mentioned above, the copper atom first lost two electrons and entered the electrolyte as an ion. The constant positive voltage, applied at the anode, then moved the copper ions toward the cathode through diffusion and electro-migration. It is evident that the mass fraction of the copper ion near the anode surface was gradually reduced. However, the mass fraction of the copper ion near the cathode surface also decreased instead of increasing, since the electrochemical reaction consumed the migrated copper ions. Moreover, the negative sulfate ions, which did not participate in the electrochemical reaction, moved in an opposite direction under the action of the applied voltage and deposited on the anode surface. As a result, the concentration of the sulfate ion closest to the cathode surface decreased, while that near the anode surface increased.

#### 5.3.1. Variation of Ion Concentration Profile without Consideration of Convection

**Figure 9:**
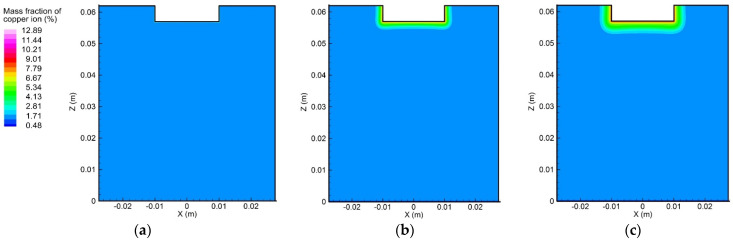
Variation of the copper ion concentration profile over time, induced by diffusion and electro-migration: (**a**) *t* = 10 s, (**b**) *t* = 300 s, and (**c**) *t* = 600 s.

**Figure 10:**
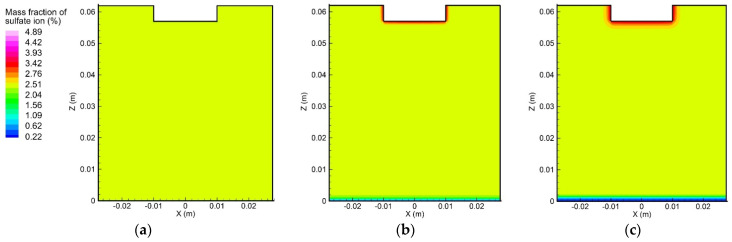
Variation of the sulfate ion concentration profile over time, induced by diffusion and electro-migration: (**a**) *t* = 10 s, (b) *t* = 300 s, and (**c**) *t* = 600 s.

In order to understand the influence of convection on the ion transport during the electrolytic process, an initial step was to study the ion movement caused solely by the diffusion and electro-migration, as exhibited in Figure 9 and Figure 10. From the initial condition, i.e., uniform concentration distribution, the concentration gradient of copper and sulfate ions gradually formed near the anode and cathode surfaces, which could be attributed to the effects of diffusion and electro-migration.

**Figure 11:**
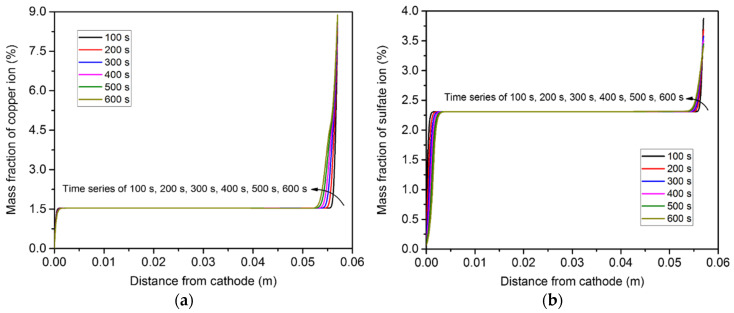
Ion concentration distributions along the centerline of the domain, induced by diffusion and electro-migration: (**a**) copper ions; (**b**) sulfate ions.

Figure 11 shows the distribution of copper ion concentration along the centerline of the container. It can be seen that the concentration gradient only existed in the vicinity of the two electrode surfaces. Furthermore, the magnitude of the concentration gradient of the copper ion near the anode surface was much greater than that near the cathode surface. Due to the electrochemical reaction, more copper ions accumulated in the top region than in the bottom region. The sulfate ion concentration profile was different from that of the copper ion, because the sulfate negative ion migrated from the cathode to the anode and was not involved in the electrochemical reaction, as mentioned above. The magnitude of the concentration gradient of the sulfate ion at the cathode surface was greater than that at the anode surface.

**Figure 12:**
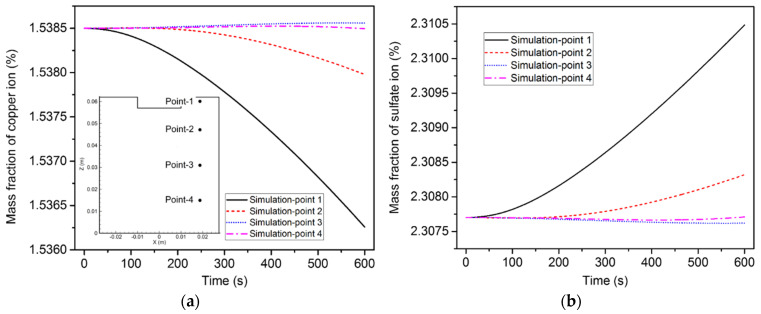
Variation of ion concentration over time at the four points, induced by diffusion and electro-migration: (**a**) copper ions; (**b**) sulfate ions.

Figure 12 illustrates the change in ion concentration over time at the four points. The copper ion concentrations at points 1 and 2 continuously decreased over time, while that at points 3 and 4 slightly increased. As for the sulfate ion concentration, it increased over time at points 1 and 2 and somewhat dropped at points 3 and 4.

#### 5.3.2. Variation of Ion Concentration Profile with Consideration of Convection

**Figure 13:**
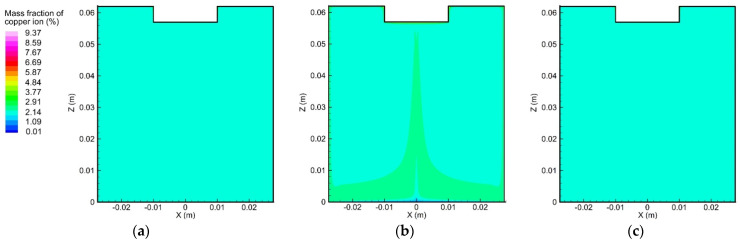
Variation of the copper ion concentration profile over time, induced by diffusion, convection, and electro-migration: (**a**) *t* = 10 s, (**b**) *t* = 300 s, and (**c**) *t* = 600 s.

**Figure 14:**
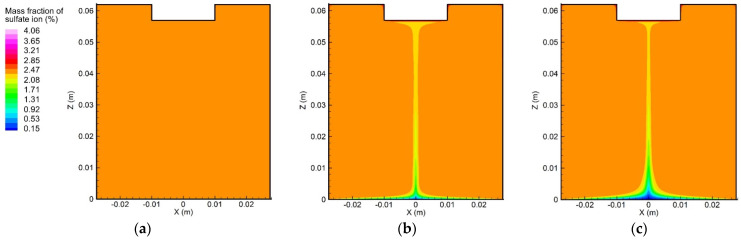
Variation of the sulfate ion concentration profile over time, induced by diffusion, convection, and electro-migration: (**a**) *t* = 10 s, (**b**) *t* = 300 s, and (**c**) *t* = 600 s.

Figure 13 and Figure 14 demonstrate the two ion concentration profiles under the effects of convection, diffusion, and electro-migration at different instants. Compared with Figure 9 and Figure 10, it is possible to infer that the flow of the electrolyte significantly affected the distribution of the ion concentration. The vortex flow first took the copper and sulfate ions away from the anode and then brought them down along the container wall to the cathode. Afterward, the copper and sulfate ions moved from the center of the cathode to the center of the anode along with the upward flow. The fluid flow was considered to reduce the ion concentration around the anode, as well as the rate of the electrochemical reaction occurring on the anode surface. The largest copper ion mass fraction in Figure 9 was about 12.89% and was found at the anode surface. However, the largest copper ion mass fraction in Figure 13 was reduced to 9.37% and was also observed at the anode surface. In addition, the distribution of the copper ion mass fraction at the cathode surface became nonuniform, decreasing from the outer edge to the center. Similarly, the largest sulfate ion mass fraction was reduced from 4.89% to 4.06%.

**Figure 15:**
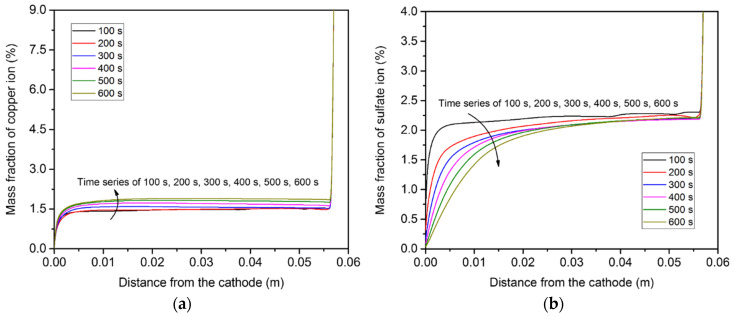
Ion concentration distributions along the centerline of the domain, induced by diffusion, convection, and electro-migration: (**a**) copper ions; (**b**) sulfate ions.

Figure 15 displays the distribution of copper ion concentration along the centerline of the container. Note that the ion concentration at the anode surface dramatically decreased in comparison to the concentration of the bulk electrolyte in a very short distance, and note that the change in the ion concentration profile along the centerline over time became more significant because of the convection.

**Figure 16:**
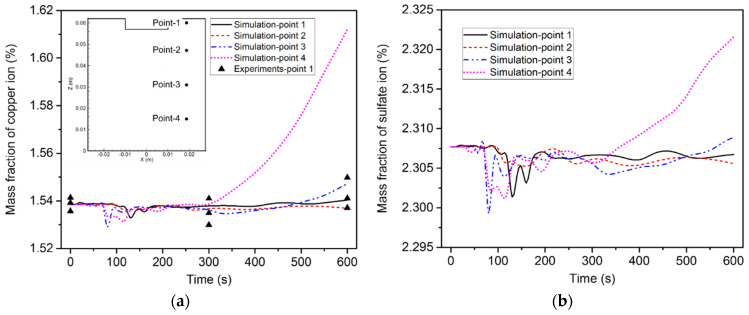
Variation of ion concentration over time at the four points induced by diffusion, convection, and electro-migration: (**a**) copper ions; (**b**) sulfate ions.

**Figure 17:**
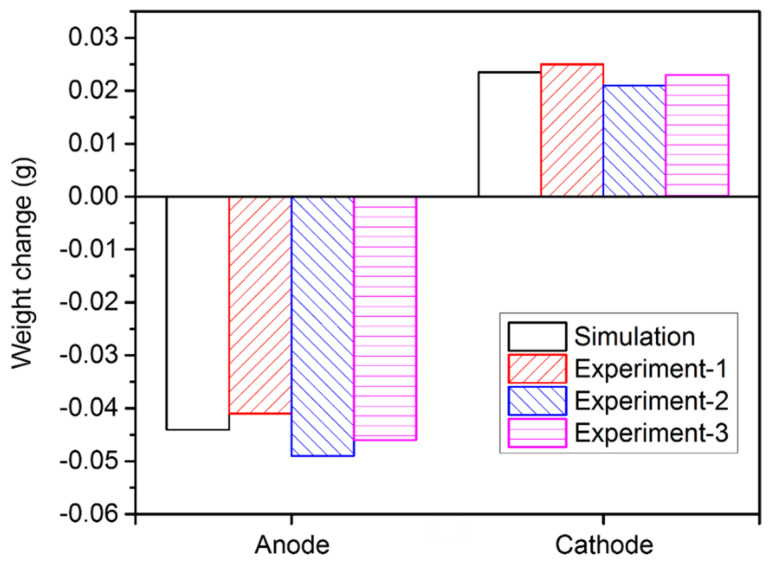
Comparison of the electrode weight change between the experiments and simulation.

Figure 16 shows the variation in the ion concentration at the four points. It is obvious that the variation trend of the ion concentration under the action of diffusion, convection, and electro-migration was much different from that under the action of diffusion and electro-migration. Since the influence of the flow became more pronounced, the ion concentration started to fluctuate after 60 s. In addition, the ion concentration of point 4 rapidly increased after 300 s, while the ion concentration of the other three points increased only slightly. Here, the calculated copper ion concentration was compared with the measured data. The experiment was repeated three times. Experimental samples were taken at a shallow depth of electrolyte, near point 1. The copper ion concentration was obtained by analyzing the sampled electrolyte at 0 s, 300 s, and 600 s. The results show that the simulated copper ion concentration matched with the corresponding measured data within an acceptable range of accuracy. The comparison of the electrode weight change between the experiments and the simulation was also measured, as illustrated in Figure 17. The mass decrement of the anode was larger than the mass increment of the cathode, due to the residual copper ions in the electrolyte.

## 6. Conclusions

In order to understand the influence of diffusion, convection, and electro-migration on ion transport during the electro-refining process, a transient three-dimensional coupled numerical model was established. The PNP equations were used to define the ion transport in the bulk of the electrolyte, while the NS and the energy equations were employed to describe fluid flow and heat transfer within the electrolyte. In addition, the kinetics of the electrochemical reaction was represented by the BV formulation. The current work aimed to establish a novel mathematical model to describe the ion transport process considering the electrical field, fluid flow, heat transfer, and electrochemical reaction during the electro-refining process. The solutions to the above equations were implemented by the finite volume method. The comparison between the measured and simulated data indicates that the numerical model can predicate ion transport with acceptable accuracy.

This research shows that, with increased heat, the temperature of the electrolyte around the anode tip gradually increases, resulting in the ascension of the electrolyte. The electrolyte then moves along the radial direction toward the outer edge of the container and, from there, down the container wall. The downward streams turn around when they reach the container bottom and then converge at the center, develop a larger stream, and finally move upward. The electrolyte heats up with the electrolytic process, and the temperature of the upper electrolyte is higher than that of the electrolyte in the lower part. Under the action of diffusion and electro-migration, the positive copper ion moves from the anode to the cathode, while the negative sulfate ion migrates in the opposite direction. However, ion movement is significantly altered if the flow is included. The vortex flow first takes the copper and sulfate ions away from the anode and then brings them down along the container wall to the cathode. Afterward, the copper and sulfate ions move from the center of the cathode to the center of the anode along with the upward flow. This flow reduces the ion concentration around the anode and results in a decreased rate of the electrochemical reaction at the anode surface. The results show that convection has a significant effect on the ion transport process, which cannot be ignored in the simulation.

The comprehensive mathematical model presented in this work delineates ion transport with the consideration of the electrical field, fluid flow, heat transfer, and electrochemical reaction during the electro-refining process. More investigations for the optimization of the existing electro-refining configurations and electrochemistry parameters can be carried out using this model, so as to improve the economic performance of the electro-refining process. Furthermore, the proposed computational framework offers a valuable basis for future research and development in the field of scrap-metal recycling technology.

## Data Availability

Data sharing is not applicable to this article.
